# Approaches to R education in Canadian universities

**DOI:** 10.12688/f1000research.10232.1

**Published:** 2016-11-30

**Authors:** Michael A. Carson, Nathan Basiliko

**Affiliations:** 1 Department of Biology and the Vale Living with Lakes Centre, Laurentian University, Sudbury, Canada

**Keywords:** R, RStudio, open source, higher education, statistics, data analysis, programming

## Abstract

*Introduction: *R language is a powerful tool used in a wide array of research disciplines and owes a large amount of its success to its open source and adaptable nature. The popularity of R has grown rapidly over the past two decades and the number of users and packages is increasing at a near exponential rate. This rapid growth has prompted a number of formal and informal online and text resources, the volume of which is beginning to present challenges to novices learning R. Students are often first exposed to R in upper division undergraduate classes or during their graduate studies. The way R is presented likely has consequences for the fundamental understanding of the program and language itself; user comprehension of R may be better if learning the language itself followed by conducting analyses, compared to someone who is learning another subject (e.g. statistics) using R for the first time. Consequently, an understanding of the approaches to R education is critical. *Methods:* To establish how students are exposed to R, we used a survey to evaluate the current use in Canadian university courses, including the context in which R is presented and the types of uses of R in the classroom. Additionally, we looked at the reasons professors either do or don’t use/teach R. *Results: *We found that R is used in a broad range of course disciplines beyond statistics (e.g. ecology) and just over one half of Canadian universities have at least one course that uses R. *Discussion and Conclusions: *Developing programming-literate students is of utmost importance and our hope is that this benchmark study will influence how post-secondary educators, as well as other programmers, approach R, specifically when developing educational and supplemental content in online, text, and package-specific formats aiding in student’s comprehension of the R language.

## Introduction

The R language was developed in the early 1990s by Ross Ihaka and Robert Gentleman in an attempt to write a statistical computing language that combined desirable aspects of two other languages, Scheme ^1^ and S ^2^. For all non-developer user purposes, R is an interpreted object-oriented language that relies heavily on packages, which contain functions that users apply to their data (see Ihaka and Gentleman, 1996 ^3^ for a more through explanation of the details and thought process behind the development of R). It could be argued that the success of R was by luck or maybe design, but the choice to target usage at statisticians meant that it had a reasonably large and dedicated user base from its inception, and subsequently, it has gained attention across academic and professional disciplines ^4^. In a general sense, the concept of user-developed packages is the reason R has gained a lot of ground over other statistical software, as the broader community is given the tools and freedom to write specific code for their disciplines and research questions, which is formatted into functions and grouped into a package. These packages are then vetted by the R Core Team and made available through the CRAN repository ^5^. This flexibility and R’s social organization has led to a rapid growth of R use and the R community, which is reflected in a number of areas, including the expansion of the Core Team, an exponential increase in the number of packages in CRAN (ca. 100 in 2001 vs. ca. 7,000 in 2016), the rise of email list traffic ^6^, the number of downloads per year, and general R activity ^7^. Additionally, based on download history from CRAN, there are millions of current R users ^8^, R has had a consistent rise in Google scholar hits (SAS and SPSS are declining) ^9^, and there have been more packages added in 2015 than have existed in all of the SAS institute’s history ^9^. Taken together, these metrics indicate the rise in popularity of R, and highlight the importance of teaching the next generation of students and researchers the most applicable skills.

We are living in a time of rapid technological advancement and an age where the free sharing of ideas is becoming a standard practice ^10, 11^. Evidence of this is seen in the proven effectiveness of the open source framework, within which R is developed ^12^. For R users, open source means not reinventing the wheel every time a new problem arises. Instead they can search for packages to address specific analyses that others have written and made publically available on CRAN or through sources like GitHub ^13^ and Omegahat ^14^. The open source nature not only means that primary R resources are freely available, but that the R community at large is also willing to provide troubleshooting support, as evidenced by the multiple independent support websites (e.g. Quick-R and Cookbook for R) and community forums that address user questions and problems (e.g. Stackoverflow and R-bloggers). Thus, this means that an average user has a diverse toolset to pull from, and an even larger support community to help them accomplish their task at hand. The open source nature of R and its sharing community are two important reasons that R is gaining popularity so rapidly in many business, research, and educational sectors.

While the R language is not specifically limited to data analysis, in science, technology, engineering, and math (STEM) disciplines it is commonly used for this purpose. For example, there were approximately 35,000 scholarly articles published across all disciplines (STEM and others) in 2015 with R as the primary analysis tool, second only to SPSS, which had decreased by 25% from the previous year ^9^. This is most likely because unlike other analysis tools, R is adaptable to specific problems, while remaining versatile enough to address more common data management, analysis, and graphing needs as well; users can easily write new code or adapt other users’ code to address their specific needs. In this way R promotes an active learning process, which is proven to increase students’ performance in STEM education ^15^. Additionally R is an “all in one” environment that streamlines data analysis workflow from data management and analysis to graphical data presentation and text processing. The concept of packages is also in line with many STEM disciplines and the nature of the scientific process and dissemination, where a reader can find the exact package used by others and do a similar analysis for their study. Finally, R gives STEM users multiple options with many packages that do nearly the same thing in slightly different ways. For example if a user wants to create a general plot, that capability is in the *base* ^16^ package, but there are also options to use an array of other packages that generate plots in slightly different ways (e.g. *lattice* ^17^ and *ggplot2* ^18^). In short, R gives users options and is easily adaptable to exact tasks at hand, greatly benefiting STEM users as well as the R community at large.

The importance of programming education is becoming evident and universities have a significant role to play ^19^. R is a prime language to use in undergraduate classrooms because it is extremely versatile, free, has a large user community, is relatively easy to learn in terms of programming (see Fox 2009 ^6^), and is supported across multiple computing platforms. This means that a student could encounter R in a wide array of classes ranging from traditional statistics to, for example, an ecological modeling or bioinformatics course. The programming skills learned in one course would easily transfer to other courses, and departments could benefit by coordinating course content to better capitalize on this continuum. Along this line, R allows students to preform practical applications rapidly upon learning the language, whereas languages geared more towards software development require more base knowledge before writing more meaningful code. This means that R is a compelling language to learn for novice programmers. Furthermore a solid foundation in R better prepares undergraduate students for postgraduate education or for seeking employment in a broad range of sectors. While there are other programing languages, the overall versatility and open source nature of R means that many research institutions and cooperate entities are using R at an increasing rate. Even if R were not the primary coding language used later in a career, learning any programming language often means that a student is better equipped to enter the job market ^20^; however, most other data management and statistical programs an undergraduate is likely to encounter are a point-and-click format (e.g. Excel and SPSS), so they gain little practical coding experience.

The goal of this survey-informed study was to highlight R usage at Canadian universities, shedding light on which types of courses use R, as well as overall R training offerings at the institutional level. Additionally, we look at some of the benefits and challenges professors encounter teaching R to their students, and motivations for using R in their research programs and teaching R in the classroom. To our knowledge this is the first study to look specifically at R usage in an educational context, and thus may also help serve as a benchmark for future characterization of R usage in universities in general and Canadian universities through time.

## Methods

### Survey methods

A survey of 70 Canadian universities was conducted using Google Forms ( https://www.google.com/forms/about/) from June 1, 2016 to June 15, 2016 to estimate the number of universities offering courses that either use or teach the R. Universities were identified as recognized institutions of higher education in Canada that offer four-year degree programs. The survey was developed to specifically address how many universities offered (a) course(s) using R and in what capacity the program was used within courses. Following research ethics approval, the survey was sent to ca. 2,500 professors in Biology, Ecology, Chemistry, Statistics, Mathematics, and Computer Science departments (considered to be the most likely sources of R usage in a university). Contact information for individual professors was obtained from departmental websites at each university in May, 2016. Only full time active faculty were sent the initial request (i.e. the survey was not sent to adjunct/emeritus professors, graduate students, or technologists). Additionally, a request was made to forward the survey to any other faculty or departments that a respondent thought appropriate or had knowledge of R usage at their particular university. The survey was formatted with conditional responses and ranged from 10 to 22 questions depending on the respondents’ answers. For example, if a respondent answered “yes” to teaching R they were taken to a different section than if they answered “no” to the same question. Survey questions and a figure diagraming conditional response layout is available in Supplementary File 1 and Supplementary File 2, respectively. Following the response period, results were downloaded and analyzed to determine the extent of R usage across Canada and evaluate usage patterns.

### Data analysis

Both individual question responses, as well as combined question information, were used to evaluate R usage. For example, the response rate of universities was simply calculated by taking the number of universities with at least one respondent divided by the number of universities surveyed, while the calculation of R usage at universities was reflected by the number of universities with at least one respondent that also had at least one class utilizing R divided by the number of respondent universities regardless of R usage. All data are expressed as counts and formal statistical tests were not preformed. As with any voluntary surveying method, it must be noted that positive sampling bias is potentially a factor; meaning it is probable that respondents were at least familiar with what R is and people unfamiliar with the program were less likely to take the time to respond. All analysis and plotting was carried out in R version 3.3.1 ^16^.

### Ethics and consent

Ethics approval was granted on May 20, 2016 from the Laurentian University Research Ethics Board (REB) under REB file number 2016-04-14. Consent was obtained through a participant consent statement ( Supplementary File 3) and electronic approval, which lead participants to the survey. This information is available in Dataset 1 ^21^, and only one participant opted out of taking part in the survey.

Conditional requirements of the REB were to retain the anonymity of individual participants. To ensure this, but preserve the ability to analyze and deposit data, university name information has been removed from the dataset and replaced with number designations. Additionally, all comments or other potential individual level identifiers have been removed.

## Results

### Overall results

Of the 2,500 professors from 70 Canadian universities invited to participate, 157 responded. Of these only one participant elected not take the survey giving a total of 156 respondents. At least one response was recorded from 61 of the universities for an 87% response rate (i.e. at least one key informant per institution). Of the 61 responding universities, 65% (40) had at least one course that used R in some manner, while 36% (22) of responding universities had courses that were either specific to the R language or used it as the primary data analysis tool. Of respondents 51% used R in at least one course. Based on the courses taught by all respondents, R was used in 26% of courses in some capacity and of the courses that used R, 16% taught the R language.

### Professors who teach R

**Figure 1.**
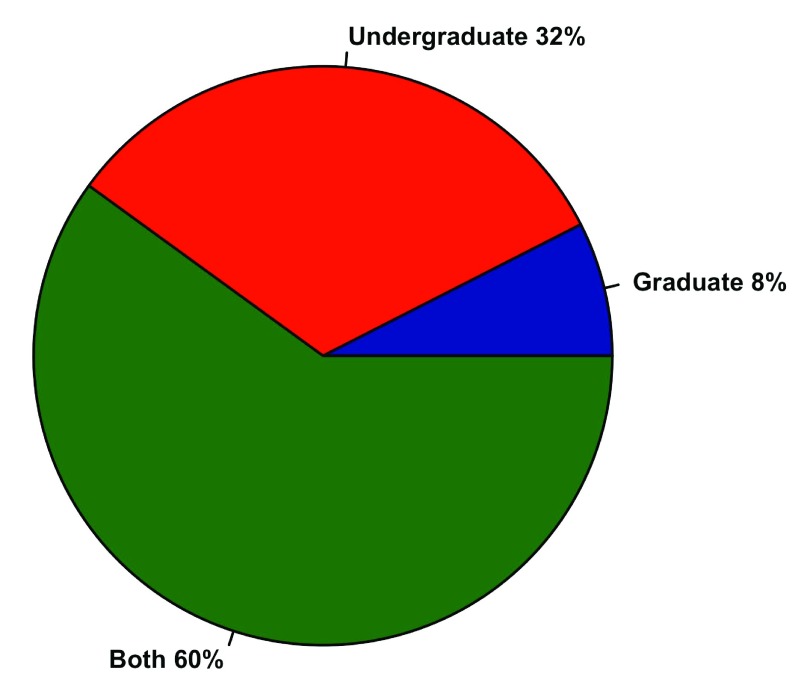
Distribution of R classes. Breakdown of course offerings for 80 professors who teach with R, where “both” means a class contains undergraduates and graduate students or the professor teaches both an undergraduate and graduate course using R.

**Figure 2.**
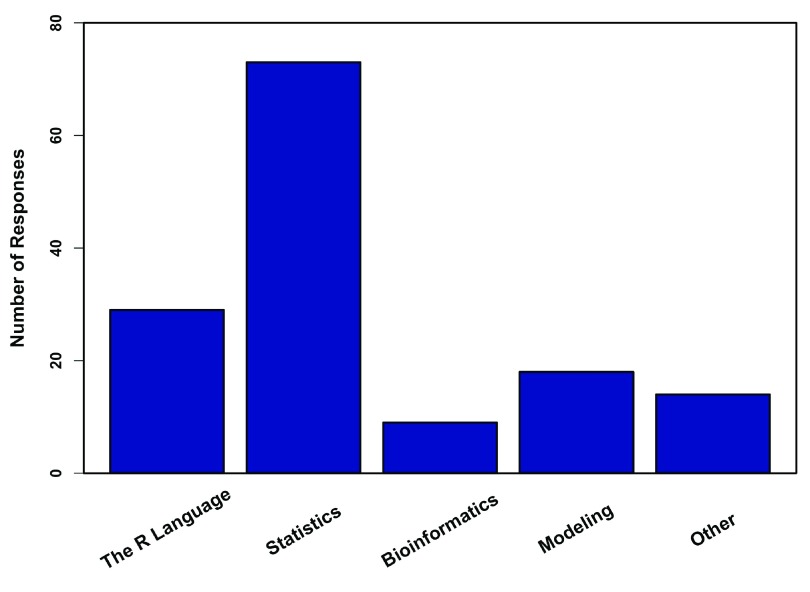
Subjects taught in R. Responses from 80 professors who teach R, regarding the subjects they teach in their courses that use R (multiple responses were allowed). Other includes climatology, population genetics, econoinformatics, and plotting.

**Figure 3.**
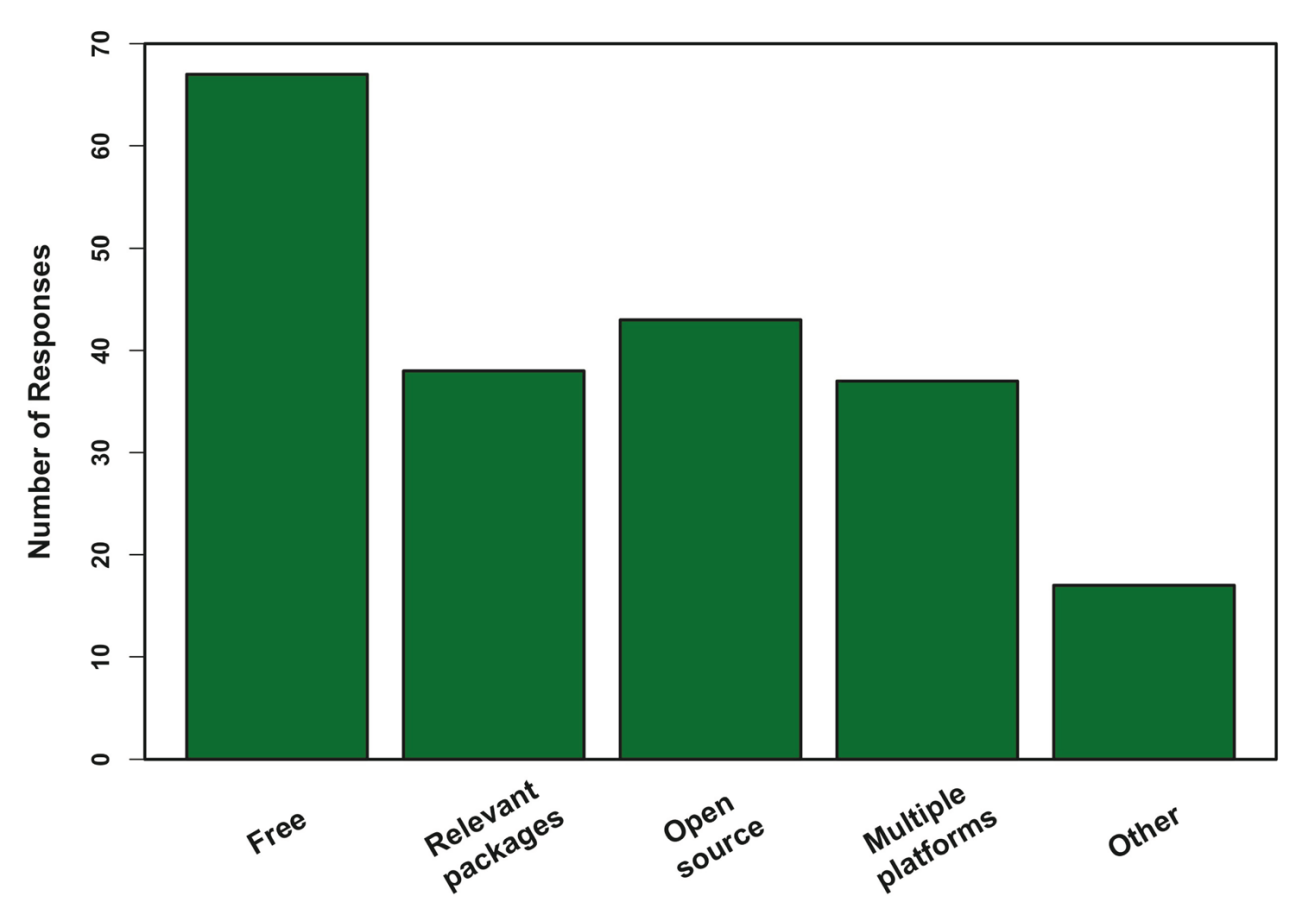
Advantages of R. Responses from 80 professors who teach R, regarding the biggest advantages to using R in the classroom (multiple responses were allowed). Other includes facilitates problem solving, teaches job applicable skills, the R community, graphics, flexibility, and reproducibility.

**Figure 4.**
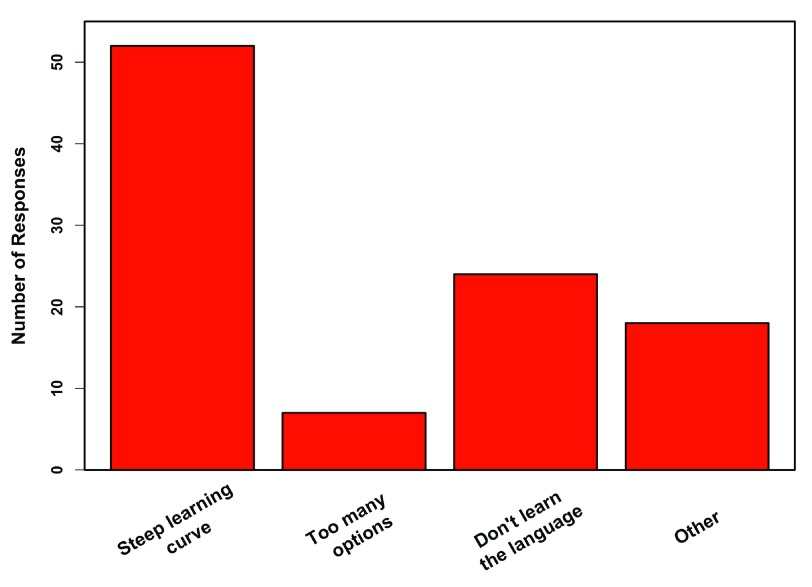
Disadvantages of R. Responses from 78 professors who teach R, regarding the biggest disadvantages to using R in the classroom (multiple responses were allowed). Other includes requires coding, colleagues cooperating, pushback from SAS users, students using multiple platforms in classroom, mainstream texts lack R examples, and R is used less in industry.

Of courses using R, 60% were offered to both undergraduate and graduate students while only 8% were graduate-only, and the remaining 32% undergraduate-only ( Figure 1). By far the most frequent use of R in the classroom was geared towards statistics, followed by courses explicitly focusing on the R language itself and ecological modeling, respectively, ( Figure 2). Professors who taught R felt the biggest advantages included that it is free, followed multiple platform support, diverse packages, and being open source; the latter three were all weighted similarly ( Figure 3). Cited disadvantages to teaching R were dominated by a steep learning curve, followed by the students not actually learning the language itself (e.g. using code that is “plug and play” and not written or altered by students; Figure 4).

### Professors who do not teach R

**Figure 5.**
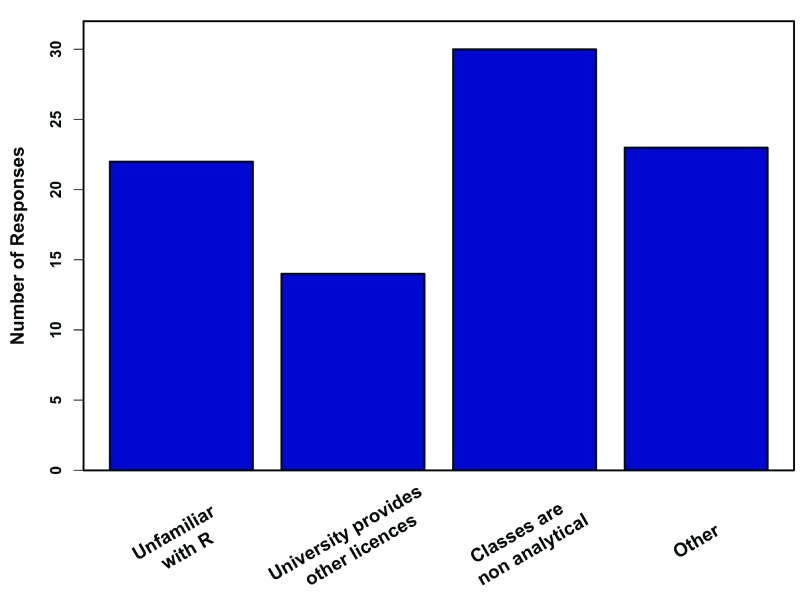
Reasons to not teach R. Responses of 76 professors who don’t use R in any classes (multiple responses were allowed). Other includes time restrictions and classes that are already using other stats programs with limited departmental cooperation on switching over.

**Figure 6.**
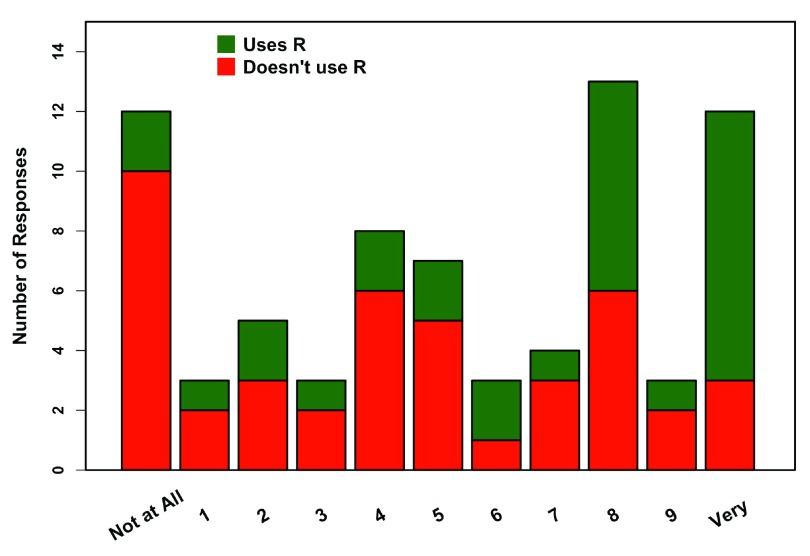
Openness to teaching R. Responses of 73 professors who don’t use R in any classes, regarding their willingness to use R in future classes. Groupings are by professors who use R in their research, but don’t teach it (green), and those who don’t teach or use R themselves (red).

A total of 76 professors did not teach with R at all. The most common reasons for not teaching with R are presented in Figure 5. Key reasons for not teaching R included teaching non-analytical courses or being unfamiliar with R. Many of the “other” responses included what could be classified as “departmental issues” (e.g. lack of time, perceived difficulty of learning R vs. programs like Excel, cooperation in coordinating between courses/professors). Professors who used R in their own research, but don’t teach R, were more open to teaching R in the future when compared to professors who were unfamiliar with R ( Figure 6). Overall, the majority of professors were open to teaching a class using R in the future.

### Professors who use R themselves

**Figure 7.**
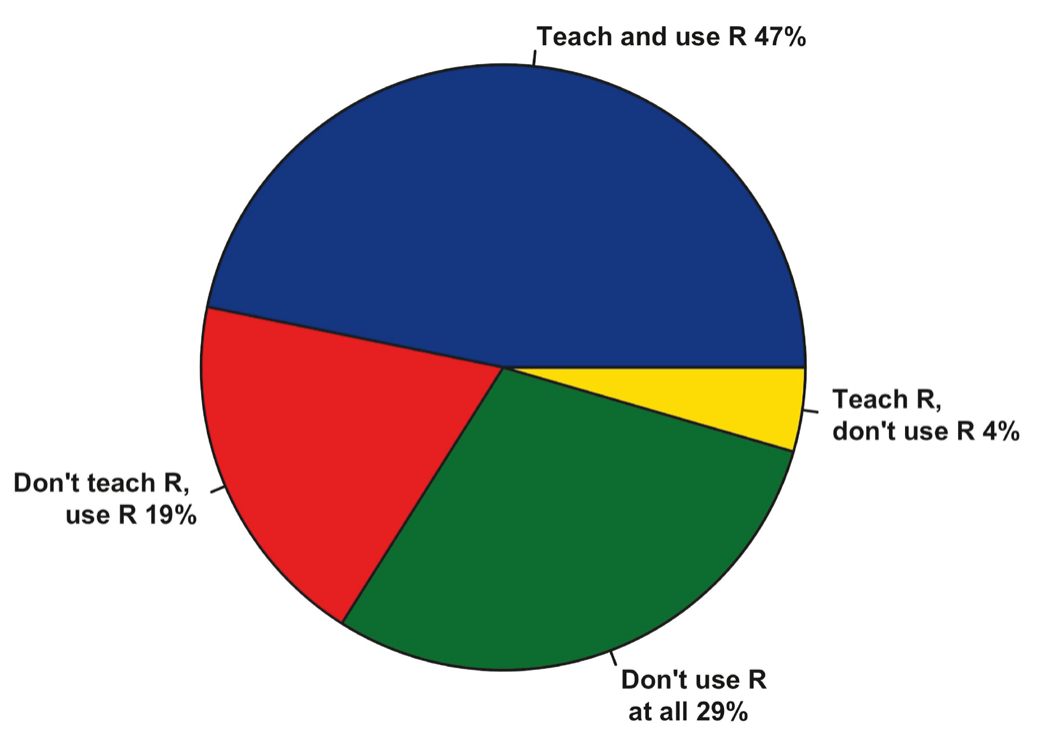
Professor R usage. Summary of how 156 professors interact with R. Note the large portion of professors who don’t teach R, but use it in their own research.

**Figure 8.**
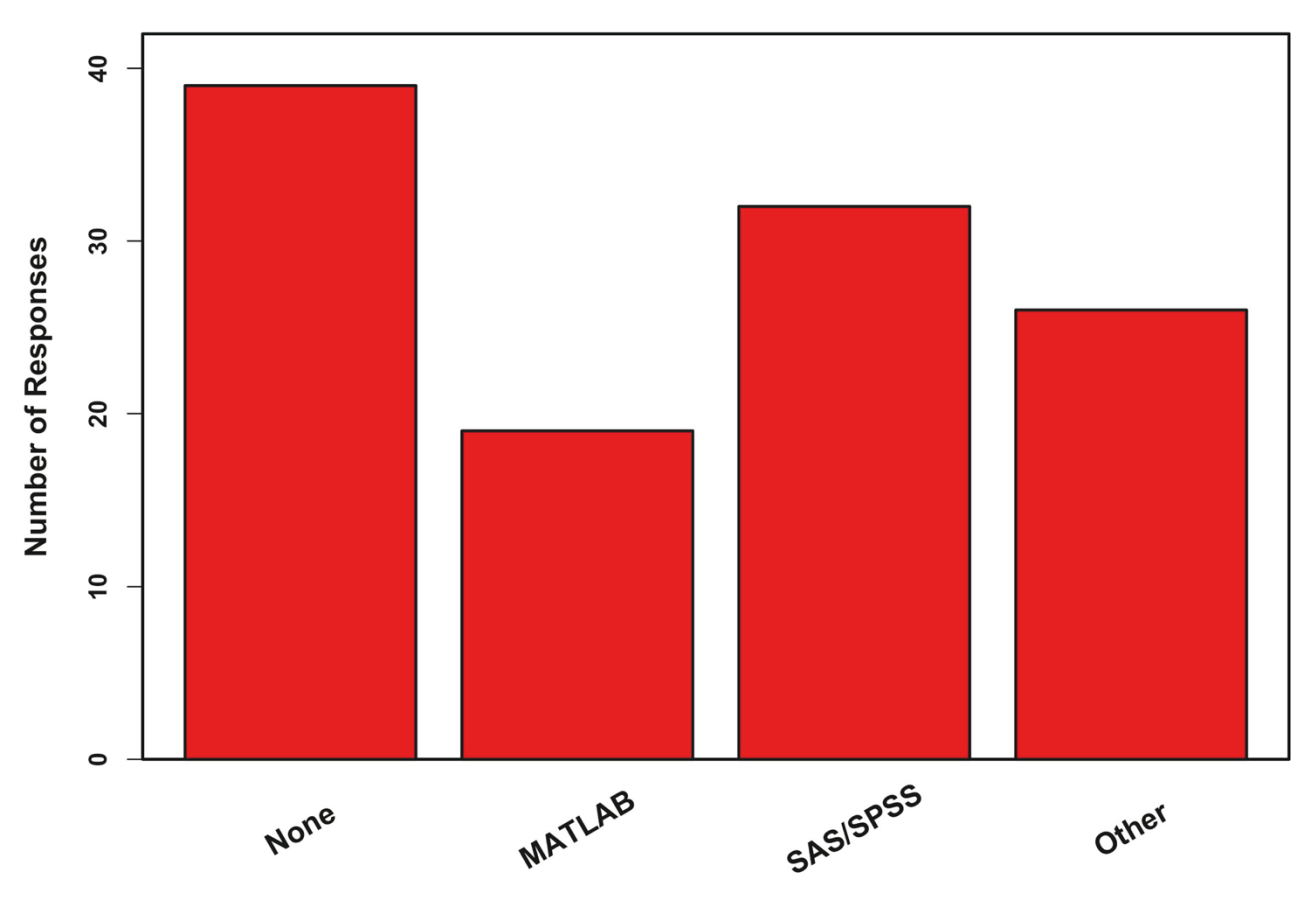
Use of other programs. Response of 100 professors who use R (could have multiple answers). Other includes Excel, LINDO, BMDP, Prism, PAST, MEGA, Statistica, Sigmaplot, Stata, JMP, DataDesk, Systat, STAN, OpenBUGS, Minitab, Mathematica.

**Figure 9.**
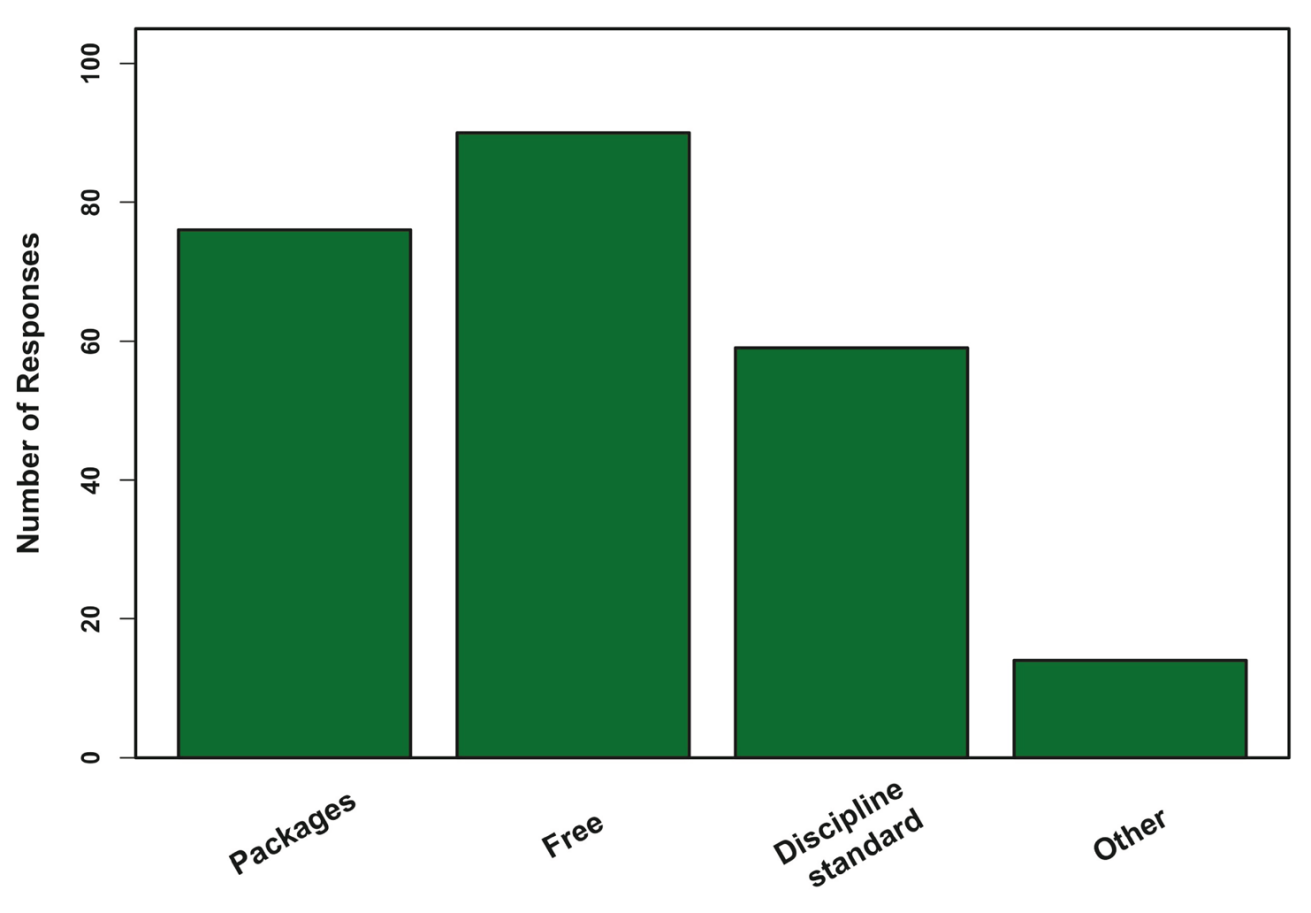
Why use R? Reasons 103 professors use R themselves (multiple responses allowed). Other includes new code/package development, multiple platform support, and user configuration/flexibility.

**Figure 10.**
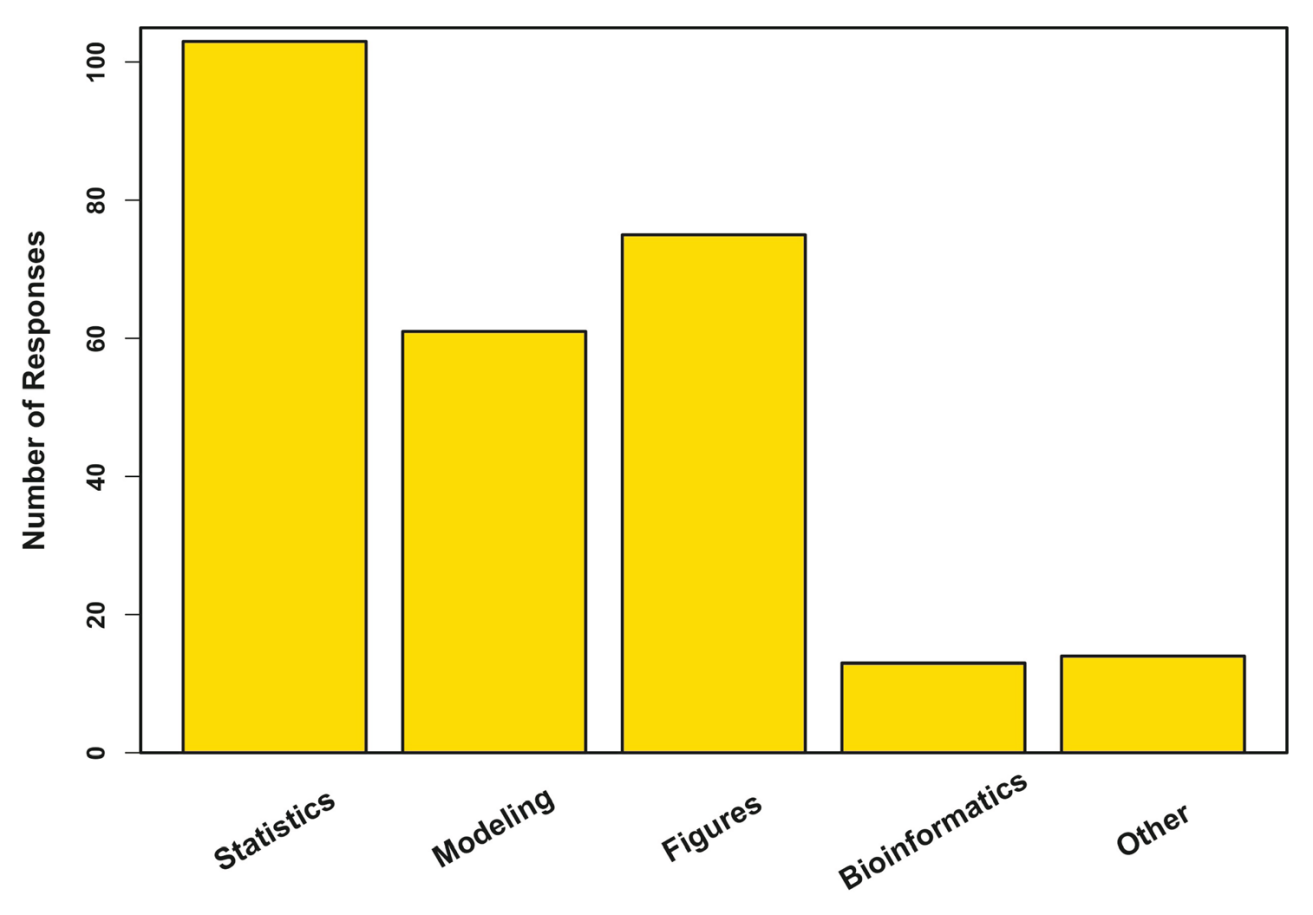
How R is used. Uses of R for 103 professors. Other includes data manipulation, simulations, and data exploration.

Professor usage of R in research did not clearly reflect them teaching (with) it in the classroom. Figure 7 shows four groups based on whether professors taught and/or used R themselves in their research. The majority of professors (66%) used R themselves, while only 51% of professors actually taught R. In total, 19% of professors who used R themselves did not teach it. Professors who used R tended to use only R, but SAS/SPSS and MATLAB were also used along with an assortment of other programs ( Figure 8). In comparison to reasons to teach with R, professors who used R still felt it being free was a good reason to use it, but also placed more emphasis on packages and it being a discipline standard ( Figure 9). All professors who used R (100%) did so for descriptive statistical analyses, while modeling and figure generation were other common uses ( Figure 10).

### R by subject area

Of the 156 respondents 154 indicated a department affiliation grouped into biology/life sciences, math/statistics, and others, including professors who had multiple appointments in biology, math, stats, and/or were in completely unique departments, e.g. decision sciences. A total of 64% of respondents were in the biology/life sciences, and 48.5% taught R. Professors who identified with math and stats departments made up 27.5% of respondents and 56% taught R. Of professors who were in statistics alone, 100% taught R in at least one course. The remaining 8.5% of respondents were categorized as “others” and 54.5% taught at least one class using R.

R survey results Click here for additional data file. Raw data from the survey questions with the university names converted to numbers and other potential respondent identifiers removed. Copyright: © 2016 Carson MA and Basiliko N 2016 Data associated with the article are available under the terms of the Creative Commons Zero "No rights reserved" data waiver (CC0 1.0 Public domain dedication).

## Discussion

The R language is beginning to make its way into Canadian universities with a wide range of courses spanning both graduate and undergraduate levels already in place. Over half of Canadian universities offer at least one course that uses R, but these courses are often not geared at the R language specifically, greatly diminishing the benefits to students. While a number of universities did offer multiple classes that use R, this was the exception and not the norm, indicating that R is not being adopted by professors and expanding throughout Canadian universities as fast as it perhaps should be. There appeared to be a positive sampling bias towards people who use R themselves; meaning a professor who was unfamiliar with R was unlikely to respond to the survey, however this is not uncommon in surveys of this type ^22^. Taking this into consideration, it is likely that these results represent the current state of R usage at Canadian universities relatively well. There was a diverse range of professor’s experience with R as well as the subjects being taught using R. This reflects common trends in the R community where the language has been adapted beyond a statistical tool for use in an array of applications, for example interactive maps (rMaps package) and developing applications ^23^. Taken together, both professors who currently teach and those who do not teach R need to consider new ways to adapt their coursework to include R in interactive and engaging ways.

By far the most common application in the classroom was statistics, which is likely due to the origins of R being geared at that community ^3^. Bioinformatics usage was a less common theme, but this is an area that will likely see significant growth in the coming years with a large amount of new package development promoted by Bioconductor (collection of packages specific to bioinformatics usage) prompted by the drop in DNA sequencing cost and rapid increase in sequence data being produced ( NCBI). While the number of courses taught that explicitly teach the R language is perhaps lower than ideal, it must be noted that courses dedicated specifically to the R language may be a lofty goal, and incorporating R into courses in any manner is a useful learning exercise. This is also in line with the general need of more computer literate students regardless of academic discipline ^24^. Overall, there weren’t many professors that responded who didn’t teach or use R themselves. The most common reason for not using R was that their classes were non-analytical. This appears justifiable, as some subjects rely less on data management and analysis. However, large portions of professors in this category were totally unfamiliar with the capabilities of R and it may be that they don’t realize that R and packages within R are not exclusively focused on descriptive statistical analyses. For example, modeling transmission of a pathogen in a virology class or movement of animals across an ecosystem in ecology classes could both be incorporated in labs in these courses using R. The importance of adapting course material to match current trends in technology is highlighted in other research that retrospectively are easy to understand the importance of early adoption into the classroom ^25– 27^. For example, the broad movement from handwriting to typewriters to computers or the change from film to digital cameras and the finer resolution examples of software, which is updated on a more frequent basis. While preservation of older technologies is important, keeping students at the cutting edge of technologies and the programs/systems that operate them are key to current education. Along this same line the most concerning reasons for not teaching R included time restrictions and/or limited departmental cooperation, as well as general apathy towards adapting course material ^28^. To us, these are potentially poor excuses for not altering courses to expose their students to a useful, widely accessible tool and emphasize a general lack of professor engagement, which is detrimental in the classroom ^29^.

Bringing R into the classroom has a number of advantages. First, it is free, so does not strain student or department budgets and is compatible with multiple platforms (Mac, PC, Linux) allowing students to download it on their personal computer instead of having to do assignments on university computers with restricted licenses. Second, it is also open source and has a large support community online with a number of forums to address virtually any sort of problem (e.g. Stackoverflow). Third, a major advantage to students is the current applicability of R in the classroom and beyond. The near exponential growth of R ^6, 7^ highlights the importance of learning the language and is indicative of a desirable skillset across academic disciplines and career paths. This is due in part to the adoption of R in many areas outside of academia, but also because R (and coding languages in general) is a skillset that many employers look for in a potential employee. That is to say learning to code is desirable for today’s students largely due to the fact that coding is a skill that is transferrable between languages and a process that teaches critical thinking and problem solving ^20, 30, 31^. So even if a student never codes again, the process of learning to code may benefit the way they approach future work. It is worth noting that with the advantages come some disadvantages, the largest being a “steep learning curve”. However, as sociologist John Fox ^6^ points out this is really in comparison to the point-and-click types of software that students are used to. In reality R is a relatively easy coding language to learn once the basic conventions are mastered, making it accessible to novice programmers.

The feasibility of introducing R into the classroom is highlighted in our study by the fact that many professors who don’t teach R are open to teaching it in the future. Furthermore, it is possible to teach classes in R even if the teacher does’nt use it themselves, and we showed that a number of professors who don’t use R themselves already teach R. After all there are numerous other skills professors pass on to students that they themselves don’t use on a regular basis, if at all (e.g. a professor teaching an introductory course would typically only research on a very small subset of what they teach). Of particular interest are the professors who use R themselves, but don’t teach R. This group could be a catalyst for universities and/or departments to introduce R into course material, greatly expanding the number of courses offering R and the subject areas using R. Willingness of adopting new technologies in the classroom is a common hurdle ^32^, but fortunately many professors in these positions are open to teaching R in the future, they just need to find the motivation to bring new material into their classroom ^28^. Admittedly it takes time and effort to adapt a class that is already “refined” and it can be difficult to be the first to take that step within a department or institution ^28, 33^, but professors should realize that the benefits greatly outweigh the costs, and can take the time to gradually begin to incorporate R into their course content. For example, a professor could promote R over other “less useful” programs (e.g. Excel), even if R will only be used for minor assignments, such as mean calculations and basic plotting. Then expansion of material could be done incrementally throughout the semester from the student’s perspective and across multiple years of lectures from the professor’s perspective. Additionally, professors should expand their own R knowledge and look for the new and exciting ways R is being used. R is no longer a purely “analytical” tool and lab courses could, for example, use R for lab report writing (markdown ^34^ is great for this), including all aspects of data management, plots, and text all in one file.

Individual comments provided valuable insights into problems with R in education, and the “learning curve” was a common theme amongst users and non-users both personally and for their students. As discussed before, this is in our view a misperception promoted by comparing R to “point and click” programs. While R is not as intuitive initially, once a foundation is established the subsequent adaptability and power over point and click platforms are large. Recently there has been an expansion of resources available to learn R in a fun and interactive way (e.g. Datacamp and swirl package ^35^). These could serve as useful companions to professors looking to use R in their classroom as an effective way of “outsourcing” much of the initial learning process. Furthermore, it is our general thought that the R community needs to expand the currently available startup material to get people familiarized with R in a more interactive way. More specifically we feel that the R education community would greatly benefit from a more centralized location for material related to course content and examples of lesson plans that incorporate R. While some examples of this are available through sources like GitHub, these are collections of individual educators and there is no comprehensive location for educational material related to R. At an institutional level some professors suggested the idea of workshops, which are a great tool in university settings ^36^. These can range from a weekend crash course to a semester long in depth introduction, which sets students and professors that are new to R on the right path from the beginning. From our personal experience, the lead author is in a trial period of teaching an R workshop, which is open to graduate, upper level undergraduate, and faculty, using hours normally devoted to teaching undergraduate labs, which is being met with positive reviews. Other comments indicated that these less formal forms of instruction may be a way to promote R in universities, ideally leading to a broader acceptance through time.

## Conclusions

It is apparent that Canadian universities are beginning to put the R language to practice in classes with nearly 2/3 of the responding universities offering at least one course that uses R. However, fewer courses teach classes that are more specific to learning the language itself. While this is a good start to exposing students to R, it appears that Canadian universities in general are lacking R-based coursework. To our knowledge, there are no similar data for R usage at universities in other countries, but a comprehensive understanding of R usage in all levels of academics is necessary and would provide critical insights. Future work could use surveys to identify broad R usage trends as we did, but would benefit even more from obtaining detailed information from syllabi or course material itself. Surveys do depend on people’s willingness to participate so perhaps individual case study reports from departments or individual teachers who have incorporated R might be of use, encouraging others to put forth the effort and use R in the classroom. Based off broad data on downloads and references to R, it is apparent that R is rapidly becoming a programming and data analysis language of choice for researchers, academics, and in industry. With this in mind it is in an institutions’ and students’ best interest to promote R in coursework among all of the STEM disciplines. Furthermore, the only “cost” to a university, department, or educator is the time required to rework course material into the R language. While this takes initial effort, we feel that the long-term benefit to students greatly outweighs this initial input. The R community is rapidly developing more “user friendly” graphical user interfaces and will continue to be at the forefront of data analysis and presentation for the foreseeable future. Without doubt, an understanding of R will benefit students beyond their coursework in postgraduate and professional settings.

## Supplementary material

**Supplementary File 1. Survey questions.** A list of all questions that participants could have been asked. Some questions are repeated for the conditional response survey. See Supplementary File 2 for question pathways.

Click here for additional data file..

**Supplementary File 2. Question flow diagram.** Diagram showing potential survey “paths” with conditional responses, numbers correspond to questions in Supplementary File 1.

Click here for additional data file..

**Supplementary File 3. Participant consent statement.** The full consent statement that participants agreed to prior to taking the survey, approved by the REB.

Click here for additional data file..

## Data availability

The data referenced by this article are under copyright with the following copyright statement: Copyright: © 2016 Carson MA and Basiliko N

Data associated with the article are available under the terms of the Creative Commons Zero "No rights reserved" data waiver (CC0 1.0 Public domain dedication). 

**Dataset 1: R survey results.** Raw data from the survey questions with the university names converted to numbers and other potential respondent identifiers removed. DOI: 10.5256/f1000research.10232.d144345 ^21^
