# Supplementary material for: Approaches to R education in Canadian universities
Source: F1000Res. 2016 Nov 30;5:2802. [Version 1] doi: 10.12688/f1000research.10232.1 (PMC5166589; doi:10.12688/f1000research.10232.1)
Supplement: Supplementary file 2 [file f1000research-5-11021-s0001.tgz › b7649dd5-4d52-4878-a779-8caeecd2f7bc.docx]

**Survey questions**

1. Consent Statement (SI 1)
2. What University are you affiliated with?
3. What department are you in?
4. Are you the department head/chair?
5. How many undergraduate courses does your department offer?
6. How many graduate courses does your department offer?
7. How many undergraduate courses do you teach?
8. How many graduate courses do you teach?
9. Do you use or teach R in any of your courses?
10. Do you have a class specific to teaching the R language?
11. What percentage of your classes use R in any capacity?
12. What subjects do you teach in R?
13. Do you use R for graduate or undergraduate classes?
14. What is the biggest advantage of teaching with R?
15. What is the biggest dis-advantage of teaching with R?
16. Do you use R for your own research?
17. Why don’t you use R in your classes?
18. How open would you be to teaching R or using R in a class in the future?
19. Do you use R for your own research?
20. How many years have you used R?
21. What do you use R for?
22. Why do you use R?
23. What other statistical programs do you use?
24. Do you have any additional comments?
25. Would you like a summary of the survey results?
