# Supplementary figures and images for: Approaches to R education in Canadian universities
Source: F1000Res. 2016 Nov 30;5:2802. [Version 1] doi: 10.12688/f1000research.10232.1 (PMC5166589; doi:10.12688/f1000research.10232.1)

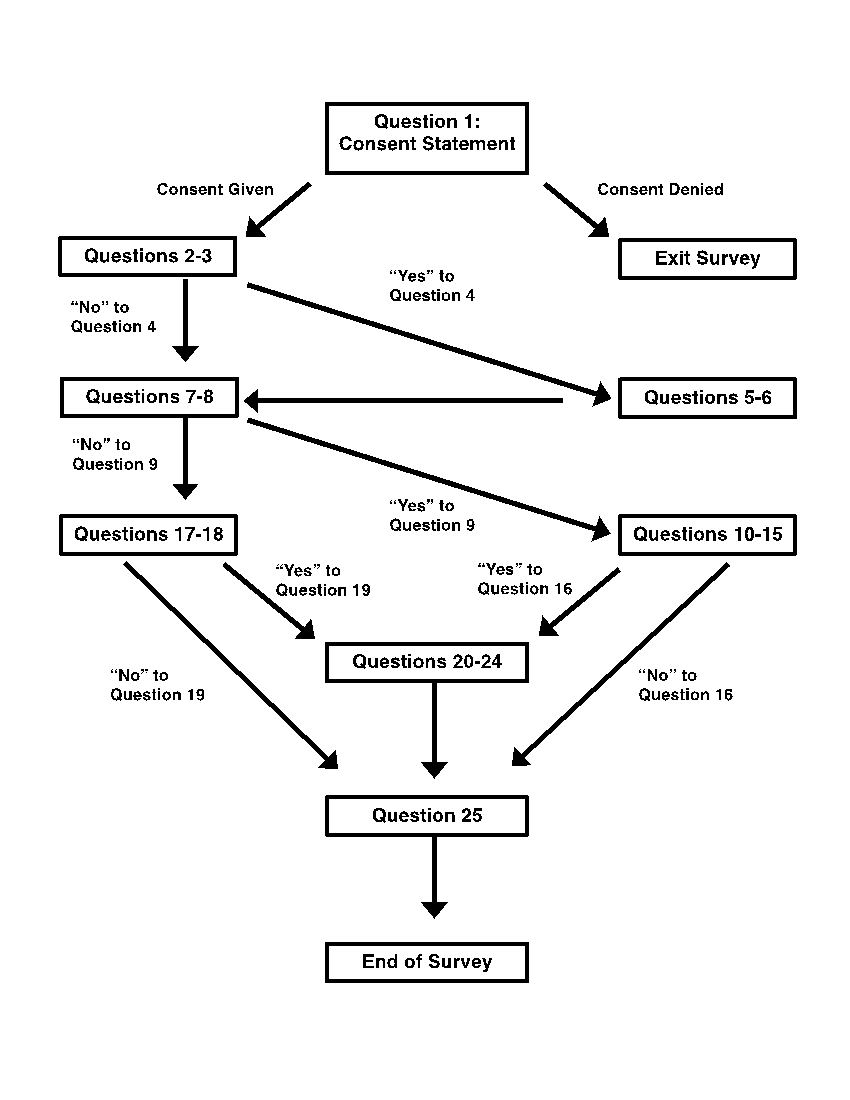

Supplement: Supplementary file 3 [file f1000research-5-11021-s0002.tgz › c570c970-6154-40a1-a969-1748eec6c61f.png]
