# Supplementary material for: Approaches to R education in Canadian universities
Source: F1000Res. 2016 Nov 30;5:2802. [Version 1] doi: 10.12688/f1000research.10232.1 (PMC5166589; doi:10.12688/f1000research.10232.1)
Supplement: Supplementary file 4 [file f1000research-5-11021-s0003.tgz › ae5a9fa8-571a-4bcf-86c6-937f52e58111.docx]

**Participant Consent Statement:**

You are invited to take part in a survey that will be used in a presentation entitled “Approaches to R education in Canadian Universities”, given at the UseR! conference in Stanford, CA, USA June 26, 2016. This survey should take no more than 5 minutes, and is *anonymous*, *voluntary,* and *confidential*. There are no risks to you and you have the right to withdraw all answers or skip any questions at any point, for any reason, with no consequence.

Data will be used to evaluate the extent to which the open source computer program R is used in Canadian Universities and the approaches to using R in the classroom. Information from this study will help inform university educators on the extent to which R is used in Canadian universities as well as the formats that R is commonly presented in. These data will be kept online until the surveys completion (June 15, 2016), will be subsequently used for presentations and publication, and will be stored indefinitely password protected files. No later than August 1, 2016 any participant or third party interested may request a full summary of the results by emailing mcarson@laurentian.ca. To protect respondent’s identities, individual responses will NOT be released at any time.

Should you have questions regarding this survey or R in general please feel free to contact the primary researcher Michael Carson (PhD Candidate in Boreal Ecology, Laurentian University) or candidate’s advisor Nathan Basiliko (CRC Tier 2 Environmental Microbiology, Laurentian University).

Email mcarson@laurentian.ca or nbasiliko@laurentian.ca

Telephone: 705-675-1151 ext. 3107or toll free at 1-800-461-4030

For questions regarding ethics of this survey please contact:

Research Ethics Officer, Laurentian University Research Office

Telephone: 705-675-1151 ext. 2436 or toll free at 1-800-461-4030

Email ethics@laurentian.ca

By checking the box below I acknowledge that I have read the Participant Consent Statement and wish to partake in the survey

Take me to the survey

I DO NOT wish to participate
