# Supplementary material for: Approaches to R education in Canadian universities
Source: F1000Res. 2016 Nov 30;5:2802. [Version 1] doi: 10.12688/f1000research.10232.1 (PMC5166589; doi:10.12688/f1000research.10232.1)
Supplement: Peer review history

# Peer review: Referee response for version 1

**DOI:** 10.5256/f1000research.11021.r18125

**Published:** 2016-12-15

**Authors:** Luc F. Bussiere

**Affiliations:** 1 Biological and Environmental Sciences, University of Stirling, Stirling, UK

This article provides useful survey data on aspects of instruction using the R programming language at Canadian Universities. The authors report intriguing data on the numbers of respondents who use R for teaching and research, the subject areas in which the respondents work, and their willingness to teach future classes using R.

 These data provide a useful glimpse of the adoption of R software in Canadian Universities, and the transparent inclusion of the survey and data makes this publication a valuable addition to the literature. My comments below are intended to provoke further critical analysis if possible.

 Although I am sympathetic to the authors’ opinions (as an instructor who uses R in my own research and teaching), I am not consistently convinced that these data support the authors’ conclusions, even though those are made somewhat tentatively. My skepticism comes from a few sources, as detailed below. I think most of my concerns could be addressed though a follow up survey and additional analyses.

 Much of the discussion is devoted to the argument that we need more teaching of R (especially in classes dedicated to the programming language itself, rather than its applications). I do not object to this assertion in principle (teaching with R has personally been a rewarding experience for me and most of my students), but the conclusion does not derive from the survey data, and the logic that underpins it is not always clear. The authors cite some pedagogical papers on the general importance of programming knowledge, but the relative value of programming per se (as opposed to its applications) for disciplines apart from computing science are not self-evident given the assumed cost to other portions of the curriculum. One could indeed use R markdown for lab report submissions, as the authors suggest on p.10, but I am not convinced that this would often be worth implementing if the main learning outcome sought is written communication skills. I think it would be useful if the authors could more clearly separate the discussion that derives directly from their survey findings from those that represent advocacy of a particular pedagogical opinion.

 As the authors acknowledge, there is a risk of positive bias in their survey because respondents unfamiliar with R may have been less likely to respond. The importance of the bias could be estimated through attempts to contact nonrespondents, and contrasts of the scores with the original surveys, and methods for computing estimates of response survey quality seem to be reasonably well established and (of course) have been developed for analysis with R ^1^. Such an effort could help clarify the importance of biases in this study.

 For a paper about a language developed explicitly for conducting statistical analyses, the lack of statistics is quite jarring. The authors draw many conclusions about differences among categories of response based on apparent patterns, but it would be quite useful to know how much confidence we should place in the relative numbers of responses. Like the analyses of survey quality mentioned above, methods for conducting multinomial models and extracting multinomial CIs are readily available within R (e.g., see Villacorta 2012), and would allow the authors to both quantify uncertainty in their proportions and illustrate confidence limits for each response measure.

 Some of the comparisons suffer from a lack of context. For example, Fig. 1 concerns the relative provision of R courses to undergraduates vs graduate students, but this contrast is difficult to interpret without more information on the number of courses in total that are offered to graduates and undergraduates. Is the rate of provision higher at the graduate level, given the smaller number of total courses on offer? I wonder if the authors can hint at the answer by assessing numbers of courses in each category at a few institutions.

 In addition to a dissatisfying lack of measures of confidence in effects, the figures are not consistently laid out to permit effective consideration of the data. For example, in Figure 6, the key response variable is a scaled measure of willingness to teach R in future classes, but that variable appears on the x-axis instead of the y. Since the most meaningful contrast is between users and non-users of R, the authors could produce a plot that illustrates the numerical response scores in the two groups (e.g., in a strip chart) along with a measure of means and confidence limits: such a presentation would support the presumed difference much more persuasively, in my opinion, than the current layout.

 Minor comments:

 I spotted a few typographic errors, including the use of the word “preform” for perform on pp. 3 and 4, and “does’nt” on p. 10.

 The Education Board that Authored citation 19 is incorrectly attributed as if it were a single author, whereas there are 8 individuals listed as authors on the report who could be acknowledged.

I have read this submission. I believe that I have an appropriate level of expertise to confirm that it is of an acceptable scientific standard, however I have significant reservations, as outlined above.

## References

[1] de HeijVSchoutenBShlomoN: RISQ manual: Tools in SAS and R for the computation of R-indicators and partial R-indicators. *Representativity Indicators for Survey Quality*.2010;

# Peer review: Referee response for version 1

**DOI:** 10.5256/f1000research.11021.r18120

**Published:** 2016-12-14

**Authors:** Eliezer Gurarie

**Affiliations:** 1 School of Environmental and Forest Sciences, University of Washington, Seattle, WA, USA

I feel odd reviewing this paper - since I have little technical expertise in assessing human survey-based studies. My interest in this topic is as a highly biased object of the study, specifically, as an enthusiastic user of R in the classroom, both for statistics and ecological modeling courses. [Is this what the animals I do research on feel like when they read my papers?]  

 Overall, the information presented is useful. The survey appears to have been conducted responsibly, with a reasonably high response rate (though with possible self-selection bias). The introduction provides a useful overview of the history and context of R's and the discussion is comprehensive and thoughtful. The availability of the survey results is a welcome contribution. 

 While the main purpose of the article seems to be to argue and advocate for the use of R in classrooms, it is not always clear how the survey results inform that argument. On the one hand, the fact that there are courses in 65% of institutions and across fields might encourage other professors to adopt R. On the other hand (given my bias) it is a shocking disservice to students that 35% of institutions use R in ZERO courses. There was a bit of a missed opportunity in that the article presents just a snapshot in time. It would have been interesting to see how the rate of R use has increased (which would have been possible by asking professors about their use of R 5 or 10 years ago). I imagine the rate of increase would have been very nearly explosive.* In any case, the claim that "R is not being adopted ... as fast as it perhaps should be" is more of an opinion (even if softly put, and one I completely agree with) than supported by the results. There are other slight disconnects between claims in the discussion and the survey, but then for this kind of pseudo-advocating article/essay this is perhaps more acceptable than what I am used to.  

 * - As a single datapoint: when I first proposed incorporating R into an introductory undergraduate statistics course at the Unversity of Washington in 2012 - not even five years ago - the idea was met with surprise and some scepticism by other instructors that the students could "hack it",  The experiment ended up being an unequivocal success, with many students claiming that was the most useful portion of the course [certainly compared to looking up t-values in a table at the back of a textbook!] and I believe is now standard in the curriculum. 

**Results and Figures:**

 The weakest point in this paper is the ugliness of the figures (which is ironic, considering that one of the main selling points of R is the ability to make beautiful graphics). I understand that the results are simple counts, but the presentation could still be improved. Figure 1 is completely unnecessary, unless it were cross-tabulated against, e.g., subject of course (an important missing bit of information), for example sorted into statistics/ mathematics/ computer science vs. life/ social sciences.  

 In almost all of the bar plots, you could use horizontal bars, ordered top to bottom from highest count to lowest count, and go ahead and include all of the "Other" categories [e.g., in figure 2, climatology, population genetics, econoinformatics, and plotting]. Those results are interesting, and there's plenty of room if you abandon the fat vertical bars. These could also be cross-tabulated and color-stacked against subject, or at least "graduate" / "undergraduate". 

 Figure 6 (though seasonally appropriate) would be much improved if it were presented as a mosaic plot (i.e.: mosaicplot(table(R.Use, Willingness))), which is much better for comparing the relative shift across categories, while reflecting the sample sizes as well. It is, incidentally, interesting that so few people answer "9" compared to "8" (I guess 9/10 of "Very" is a more slippery concept than 4/5 of "Very"!) There's a psychological effect here somewhere, but in the meantime you might be better off pooling 1-3, 4-6, 7-9.

 I must confess I would have no idea how to answer the question in Figure 10 - there is so much overlap.  I really don't see how one can separate "Modelling" (and, often, "Statistics") from "Data manipulation" "Simulation", "Data Exploration", "Visualization", etc. 

**Discussion**

 Among the tools which facilitate the use of R in the classroom, one of the most important is the use of 'knitr' and 'Rmarkdown' to easily generate documents that combine text, math, code, figures and output. This is a very important omission. Perhaps the single most practical use of "knitting" documents is for teaching material - including lectures, labs and homework assignments - in particular for learning R. Also, report generation itself is a useful and totally accessible skill to teach, particularly considering the importance of reproducibility of analysis (another important advantage of R over point-and-click tools). 

**Minor:**

 Discussion - there's a "does'nt" that should be "doesn't".

I have read this submission. I believe that I have an appropriate level of expertise to confirm that it is of an acceptable scientific standard, however I have significant reservations, as outlined above.

# Peer review: Referee response for version 1

**DOI:** 10.5256/f1000research.11021.r18124

**Published:** 2016-12-12

**Authors:** Colin W. Rundel

**Affiliations:** 1 Department of Statistical Science, Duke University, Durham, NC, USA

The authors describe the results of a survey of Canadian academics on their use of R in their courses and in their own work. While there is good information on the increasing popularity of R on the web, in industry, and in scholarly articles there is far less information on how R is being taught. It is in this important area that the paper provides some much needed insight.

 In particular, I think many researchers would be surprised to find courses being taught using R in more than half of Canada's universities. Giving other educators this kind of information is tremendously valuable in inducing other educators to also decide to make the jump to R. In particular, being able to point to other universities and courses where R is being successfully taught is a strong argument against common complaints like the learning curve being too steep. 

 While a more systematic examination of R offerings across universities would have more reliable results than this survey, I believe that it still offers valuable (if potentially slightly biased) insights into the basic patterns of R education. The authors results are very encouraging to me as a educator interested in teaching R, but they also show that there is much more we can do in promoting R at other universities as well as growing our own course offerings locally.

**Major Comments**

 Introduction

-  I think the history as stated underplays the importance of R being free software (particularly as compared to S), this aspect also is clearly a hugely influential factor for professors based on the survey results.
- *there have been more packages added in 2015 than have existed in all of the SAS institute's history* - this comparison is based the number of packages added to CRAN vs a rough estimate of the number of procs contained in SAS 9.3 (from r4stats.com). This is a weak comparison originally which is then confusingly stated in the paper.
- While touched on tangentially I think reproducible research is worth mentioning explicitly, particularly in reference to the strength of programming languages vs. point and click tools.

Results

- *Based on the courses taught by all respondents, R was used in 26% of courses in some capacity and of the courses that used R, 16% taught the R language*. I believe that this would be more interesting to see broken down based on the respondent's field. In general, additional cross tabulation by discipline would give more insight into the data.
- Figure 1 is not really needed, giving the values in the text is sufficient in my mind. Also it is somewhat confusing about which subset of classes this breakdown applies to - this is generally true for many of the other figures. See my Figures comment below.

Discussion

- *indicating that R is not being adopted by professors and expanding throughout Canadian universities as fast as it perhaps should be*. I don't entirely follow the logic here, it is not clear how to establish how fast it is or should be expanding. While I don't disagree with the sentiment, this comes across as an unsupported opinion.
- *Taking this into consideration, it is likely that these results represent the current state of R usage at Canadian universities relatively well.* Again, this comes off more of an opinion than what is supported by the survey results and a claim like this needs additional support. The later conclusions are not invalided by removing this claim.

Figures

- Most figures could be shrunk considerably without negatively affecting readability, e.g. Figs 2-5. In some cases it might improve readability to combine plots into facets within a single figure (connecting figures to subsections).

**Minor Comments**

 Introduction

- This is the first I've ever heard of omegahat, seems to be on a very different scale than github or even r-forge.
- While not without their issue, it seems worthwhile to also mention R's mailing lists and special interest groups.

Methods

- *A survey of 70 Canadian universities was conducted using Google Forms (https://www.google.com/forms/about/) from June 1, 2016 to June 15, 2016 to estimate the number of universities offering courses that either use or teach* **the** *R*

Results

- The basic organizational structure is based subsets of the respondents, it would be helpful to indicate the size of each of these subsets. For example, n=80 for professors who teach with R is only given explicitly in the Figure 2 label.
- *Professors who taught R felt the biggest advantages included that it is free, followed **by** multiple platform support, diverse packages, and being open source;*

Discussion

- *(e.g. Datacamp and **the** swirl package)*
- *Furthermore, it is our general thought that the R community needs to expand the currently available startup material **s** to get people familiarized with R in a more interactive way.*

Figures

- Bar plot labels are rotated, this is not needed - it makes the labels harder to read and takes up unnecessary space.

Conclusions 

- However, fewer **professors** teach classes that are more specific to learning the language itself.*

I have read this submission. I believe that I have an appropriate level of expertise to confirm that it is of an acceptable scientific standard.
